# Angiotensin-I Converting Enzyme (ACE) Inhibitory and Anti-Oxidant Activities of Sea Cucumber (*Actinopyga lecanora*) Hydrolysates

**DOI:** 10.3390/ijms161226140

**Published:** 2015-12-04

**Authors:** Raheleh Ghanbari, Mohammad Zarei, Afshin Ebrahimpour, Azizah Abdul-Hamid, Amin Ismail, Nazamid Saari

**Affiliations:** 1 Faculty of Food Science and Technology, Universiti Putra Malaysia, 43400 UPM Serdang, Selangor, Malaysia; ghanbari.rahele@yahoo.com (R.G.); mzarei.mail@gmail.com (M.Z.); a_ebrahimpour@yahoo.com (A.E.); azizahah@upm.edu.my (A.A.-H.); 2 Department of Food Science and Technology, College of Agriculture and Natural Resources, Islamic Azad University, Sanandaj 66131, Iran; 3 Faculty of Medicine and Health Sciences, Universiti Putra Malaysia, 43400 UPM Serdang, Selangor, Malaysia; aminis@upm.edu.my

**Keywords:** *Actinopyga lecanora*, anti-oxidative, ACE inhibitory

## Abstract

In recent years, food protein-derived hydrolysates have received considerable attention because of their numerous health benefits. Amongst the hydrolysates, those with anti-hypertensive and anti-oxidative activities are receiving special attention as both activities can play significant roles in preventing cardiovascular diseases. The present study investigated the angiotensin-I converting enzyme (ACE) inhibitory and anti-oxidative activities of *Actinopyga lecanora* (*A. lecanora*) hydrolysates, which had been prepared by alcalase, papain, bromelain, flavourzyme, pepsin, and trypsin under their optimum conditions. The alcalase hydrolysate showed the highest ACE inhibitory activity (69.8%) after 8 h of hydrolysis while the highest anti-oxidative activities measured by 2,2-diphenyl 1-1-picrylhydrazyl radical scavenging (DPPH) (56.00%) and ferrous ion-chelating (FIC) (59.00%) methods were exhibited after 24 h and 8 h of hydrolysis, respectively. The ACE-inhibitory and anti-oxidative activities displayed dose-dependent trends, and increased with increasing protein hydrolysate concentrations. Moreover, strong positive correlations between angiotensin-I converting enzyme (ACE) inhibitory and anti-oxidative activities were also observed. This study indicates that *A. lecanora* hydrolysate can be exploited as a source of functional food owing to its anti-oxidant as well as anti-hypertension functions.

## 1. Introduction

Hypertension, one of the major causes of chronic diseases worldwide, is recognized as a risk factor of cardiovascular diseases (CVDs) in developed and developing countries. The prevalence of hypertension is increasing, and it has been projected that more than 1.56 billion people worldwide will suffer from hypertension by 2025 [1]. Angiotensin-I converting enzyme (ACE) plays a crucial role in the regulation of blood pressure via renin-angiotensin and the kinin-kallikrein systems. In fact, ACE promotes the conversion of angiotensin I into the potent vasoconstrictor angiotensin II as well as inactivating the bradykinin a vasodilator [2]. The dual functions of ACE cause an increase in blood pressure and finally lead to the development of hypertension [3].

Oxidation is a very important process in aerobic metabolism, particularly in vertebrates and humans; however, it contributes to the formation of free radicals [4]. When these unstable free radicals exist in excess or cellular defenses are deficient due to absence of anti-oxidative molecules, they may damage the bio-molecules. Furthermore, free radicals released through oxidative stress also would damage nucleic acids (DNA or RNA), lipids and proteins, thereby resulting in cell death and tissue damages. Moreover, oxidative stress leads to different kinds of human disease including cancer, cardiovascular diseases [5], stroke and hypertension [6]. Although the human body has its own defense system against free radicals, it is not very effective in preventing the damage completely. Thus, foods containing anti-oxidative agents can be used to help and protect the human body against such oxidative damages [7].

Moreover, under the condition of high blood pressure, angiotension II increases the oxidative stress as it intervenes with several of its cellular actions by stimulating the formation of intracellular reactive oxygen species (ROS) [8]. Therefore, apart from controlling blood pressure, ACE inhibitors have been shown to enhance the anti-oxidative defense system in animals and humans through inhibition of the formation of angiotensin II. Thus, functional food products with multi-bioactivities are gaining wider attention. Meisel [9] reported that some protein hydrolysates are considered multifunctional as they exhibited two or more different biological activities simultaneously. For instance, some peptides and protein hydrolysates such as winged bean seed hydrolysates [10], peptide from the algae protein waste [11] and potato hydrolysates [12] possess both ACE inhibitory and anti-oxidative properties. Therefore, it could be very useful to develop functional food ingredients for controlling the CVD and oxidative stress.

*Actinopyga lecanora*, commonly known as stone fish, is classified among the edible species of sea cucumber. Due to its relatively high protein content [13], it could be a potential commercial source for generating enzymatic protein hydrolysates with multifunctional bioactivities. Thus, this study aimed to generate bifunctional protein hydrolysates with ACE inhibitory and anti-oxidative activities from *A. lecanora*. To the best of our knowledge, this is the first study reported on the aforementioned bifunctional properties of *A. lecanora* protein hydrolysates. The finding of current study can provide fundamental information for further study in this field. In addition, the bioactive peptides of *A. lecanora* hydrolysates can be used as an ingredient in functional foods, pharmaceuticals and nutraceuticals.

## 2. Results

### 2.1. Peptide Content

The hydrolysis efficiency was evaluated by determining peptide content in the hydrolysates that had been generated using six proteases. Generally, peptide content, which indicates the extent of hydrolysis, increases with increasing the degree of hydrolysis [14]. Table 1 presents changes in peptide contents as a function of hydrolysis time. The results show a significant (*p* < 0.05) correlation between the increase of hydrolysis time and the content of the peptides in all protein hydrolysates. In most of the treatments, the variation of peptide generation after 9 h of hydrolysis was almost negligible and it could be explained by the hydrolysis of peptides into amino acids [15]. Through the six proteases used, papain and alcalase showed higher proteolytic activities compared to other enzymes. The peptide contents after 24 h hydrolysis by papain, alcalase, bromelain, flavorzyme, pepsin and trypsin were 4.47, 4.40, 3.80, 2.13, 1.69 and 1.50 mg glutathione/mL hydrolysates, respectively. Alcalase has been reported to produce the highest amount of bioactive peptides from marine resources such as sardine by-product [16]. The results presented above are in agreement with the previous study on sea cucumber (*Stichopus japanicous*) that was digested using papain, pepsin, trypsin, acid protease and neutral protease [17], in which the highest yield of the peptide was obtained with papain. Therefore, peptide content increased significantly during hydrolysis, indicating that the peptides were liberated consistently during hydrolysis.

### 2.2. Amino Acid Composition

As shown in Table 2, there were significant differences (*p* < 0.05) in amino acid composition of untreated *A. lecanora* and generated hydrolysates in terms of individual amino acid and total amino acid content. The total amino acid content in the untreated *A. lecanora* was 878.91 mg/g dry weight, and it significantly decreased after 24 h hydrolysis with different enzymes. It was higher in alcalase, bromelain and pepsin generated hydrolysates, whereas the lowest amount was generated after 24 h hydrolysis by trypsin. Glycine, glutamic acid and aspartic acid, which accounted for 140.63, 106.83 and 78.83 mg/g dry weight, respectively, were the major amino acids in the *A. lecanora.* These amino acids have been reported as the main amino acids in other sea cucumber species such as *Isostichopus badionotus* [18] and *Stichopus japonicas* [19]. Although the target concentration of amino acids after hydrolysis with different enzymes was reduced, glycine, glutamic acid and asparatic acid were still the major amino acids in the hydrolysates. Moreover, Dong *et al.* [20] have reported the changes in amino acid compositions after hydrolysis of silver carp protein.

### 2.3. Effect of Enzymatic Hydrolysis on the Bioactivities of A. lecanora Hydrolysates

ACE inhibitory and anti-oxidative activities of A. lecanora hydrolysates were measured using a 1-h interval between 0 h and 24 h of hydrolysis. 

**Table 1:** Changes in peptide contents as a function of time during hydrolysis of *A. lecanora* with various proteases as monitored by the OPA assay.

Hydrolysis Time (h)	Peptide Content of Hydrolysates (mg Glutathione Equivalent/mL)					
	Papain	Alcalase	Bromelain	Flavourzyme	Pepsin	Trypsin
0	ND ^j^	ND ^j^	ND ^i^	ND ^f^	ND ^i^	ND ^g^
1	1.93 ± 0.10 ^Ai^	1.55 ± 0.02 ^Bi^	1.78 ± 0.02 ^ABh^	1.10 ± 0.02 ^Ce^	1.10 ± 0.02 ^Ch^	1.01 ± 0.06 ^Ce^
2	2.20 ± 0.06 ^Ah^	1.95 ± 0.03 ^Ah^	1.90 ± 0.05 ^Agh^	1.14 ± 0.08 ^Be^	1.11 ± 0.02 ^Bh^	1.05 ± 0.04 ^Be^
3	2.43 ± 0.07 ^Ag^	2.15 ± 0.10 ^Bg^	2.06 ± 0.07 ^Bfg^	1.17 ± 0.02 ^Cde^	1.14 ± 0.03 ^Cgh^	1.03 ± 0.03 ^Ce^
4	2.80 ± 0.02 ^Af^	2.50 ± 0.05 ^Bf^	2.17 ± 0.08 ^Cef^	1.20 ± 0.06 ^Dde^	1.23 ± 0.04 ^Dfg^	1.08 ± 0.04 ^De^
5	2.79 ± 0.04 ^Af^	2.63 ± 0.09 ^Af^	2.25 ± 0.07 ^Bef^	1.27 ± 0.06 ^Cd^	1.30 ± 0.07 ^Cef^	1.00 ± 0.00 ^Dde^
6	3.01 ± 0.06 ^Ae^	2.98 ± 0.06 ^Ae^	2.36 ± 0.05 ^Be^	1.50 ± 0.03 ^Cc^	1.40 ± 0.04 ^Cde^	1.07 ± 0.03 ^Dde^
7	3.51 ± 0.03 ^Ad^	3.33 ± 0.04 ^Bd^	2.76 ± 0.06 ^Cd^	1.61 ± 0.04 ^Db^	1.50 ± 0.03 ^Dcd^	1.21 ± 0.02 ^Ecd^
8	3.91 ± 0.03 ^Ac^	3.70 ± 0.03 ^Bc^	3.09 ± 0.07 ^Cc^	1.70 ± 0.07 ^Db^	1.54 ± 0.04 ^Dbc^	1.30 ± 0.06 ^Ebc^
9	4.10 ± 0.05 ^Ab^	3.88 ± 0.05 ^Bb^	3.41 ± 0.08 ^Cb^	2.05 ± 0.07 ^Da^	1.62 ± 0.03 ^Eab^	1.35 ± 0.02 ^Fbc^
10	4.40 ± 0.06 ^Aa^	3.90 ± 0.04 ^Bb^	3.66 ± 0.09 ^Ca^	2.10 ± 0.07 ^Da^	1.67 ± 0.02 ^Ea^	1.41 ± 0.06 ^Fab^
24	4.47 ± 0.03 ^Aa^	4.40 ± 0.07 ^Aa^	3.80 ± 0.08 ^Ba^	2.13 ± 0.05 ^Ca^	1.69 ± 0.05 ^Da^	1.50 ± 0.03 ^Ea^

Each value represents the mean ± SD of three replications; ND: Not detected; ^a−j^ Mean values within a column with different superscript letters are significantly different (*p* < 0.05); ^A−F^ Mean values within each row with different superscript letters are significantly different (*p* < 0.05).

#### 2.3.1. ACE Inhibitory Activity

**Figure 1:**
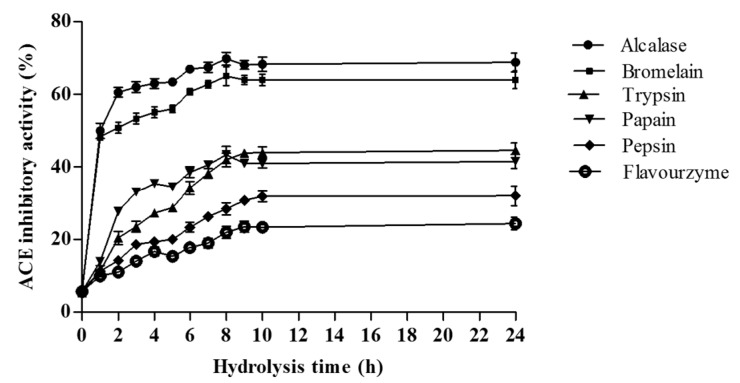
ACE inhibitory activities (%) of *A. lecanora* hydrolysates as affected by hydrolysis time using enzymatic digestion during 24 h. Sample concentration for this assay was 10.0 mg dry weight/mL. Results represent the mean ± SD of three replications.

Figure 1 shows the angiotensin-I converting enzyme (ACE) inhibitory activities of hydrolysates at a concentration of 10.0 mg of dry weight/mL. The ACE inhibitory activity of non-hydrolyzed *A. lecanora* was determined to be 6.0%, and it significantly increased upon hydrolysis (*p* < 0.05). The ACE inhibitory activities of hydrolysates generated with all six enzymes varied over a wide range from 9.90% to 69.80%.

Among the hydrolysates, alcalase and bromelain-generated hydrolysates exhibited the highest ACE inhibitory activities (69.80% and 64.50%, respectively) (*p* < 0.05) while those generated by trypsin, papain, pepsin, and flavourzyme were 44.50%, 43.40%, 32.00% and 24.40%, respectively. 

**Figure 2:**
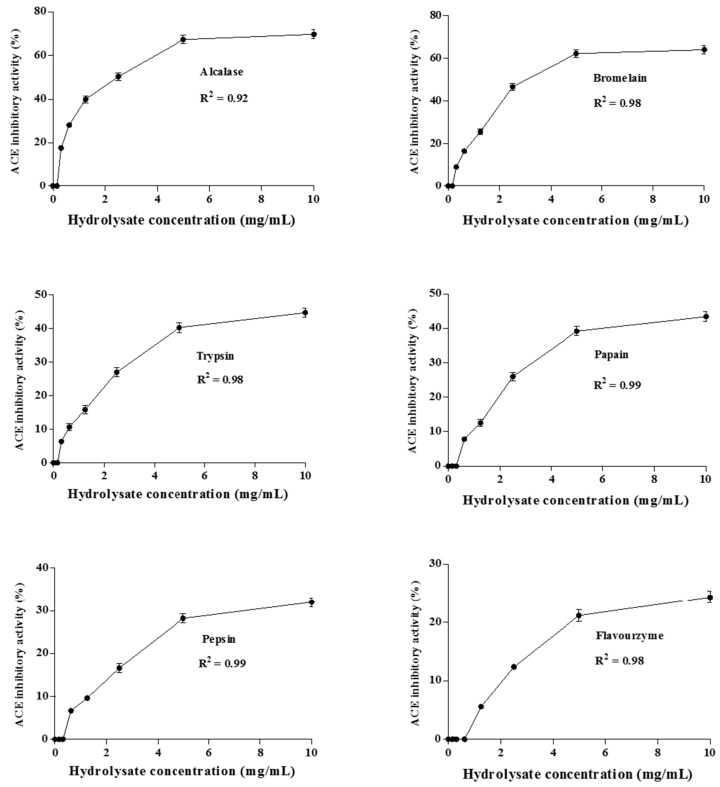
ACE inhibitory activities (%) of *A. lecanora* hydrolysates at different concentrations between 0.0 and 10.0 mg of dry weight/mL. Results represent the means ± SD of three replications.

The ACE inhibitory activity of protein hydrolysates increased with extended incubation time during the first 8 h (Figure 1). However, further digestion up to 8 h for the alcalase and papain hydrolysates resulted in a decrease in the activity, which could be a result of peptides being degraded to smaller sizes upon prolonged hydrolysis. The same finding has been reported when alcalase was used for hydrolysis of tuna liver [21] and of goby muscle [22]. Therefore, an overall increase in ACE inhibition activity with increase of hydrolysis time reflects the effectiveness of hydrolysis towards enhancing ACE inhibitory activity of the *A. lecanora* hydrolysates. The ACE inhibitory activity of *A. lecanora* hydrolysates at various concentrations (0.0–10.0 mg of dry weight/mL) was also investigated. The ACE inhibitory activities of all hydrolysates followed a concentration-dependent manner, and increased by enhancement of the concentration (Figure 2).

**Table 2:** Amino acid composition (mg/g dry weight) of freeze dried *A. lecanora* and *A. lecanora* hydrolysates after 24 h of hydrolysis *.

Amino Acid	*A. lecanora*	Papain	Alcalase	Bromelain	Flavourzyme	Trypsin	Pepsin
Aspartic acid (D)	78.83 ± 1.56 ^a^	50.33 ± 0.20 ^b^	55.96 ± 0.22 ^b^	53.16 ± 5.60 ^b^	36.07 ± 3.50 ^c^	17.27 ± 1.25 ^d^	49.21 ± 1.60 ^b^
Glutamic acid (E)	106.83 ± 3.90 ^a^	86.86 ± 1.10 ^b^	95.63 ± 1.25 ^b^	87.79 ± 6.74 ^b^	63.68 ± 4.68 ^c^	29.90 ± 2.12 ^d^	89.62 ± 1.21 ^b^
Serine (S)	34.11 ± 0.40 ^a^	23.03 ± 2.40 ^c^	27.80 ± 0.71 ^b^	28.35 ± 0.03 ^b^	18.85 ± 0.41 ^d^	7.99 ± 0.60 ^e^	20.47 ± 0.17 ^c,d^
Histidine (H)	10.93 ± 0.76 ^a^	4.30 ± 0.02 ^b,c^	5.88 ± 0.30 ^b^	4.83 ± 0.83 ^b,c^	4.55 ± 0.12 ^b,c^	1.60 ± 0.11 ^d^	3.58 ± 1.23 ^c^
Arginine (R)	65.98 ± 2.30 ^a^	45.62 ± 1.83 ^d^	56.62 ± 0.50 ^b,c^	58.64 ± 1.12 ^b^	32.73 ± 1.80 ^e^	16.55 ± 1.22 ^f^	54.40 ± 1.06 ^c^
Thereonine (T)	44.13 ± 1.43 ^a^	29.75 ± 2.60 ^c^	31.44 ± 0.80 ^b,c^	35.26 ± 1.18 ^b^	25.16 ± 1.58 ^d^	9.77 ± 0.30 ^e^	29.96 ± 0.72 ^c^
Lysine (K)	45.47 ± 2.10 ^a^	23.43 ± 4.10 ^b^	24.85 ± 1.00 ^b^	20.56 ± 1.26 ^b,c^	17.25 ± 2.83 ^c,d^	5.42 ± 0.35 ^e^	12.73 ± 0.23 ^d^
Tyrosine (Y)	44.14 ± 1.43 ^a^	29.65 ± 2.60 ^c^	31.44 ± 0.30 ^b,c^	35.26 ± 1.60 ^b^	25.16 ± 1.60 ^d^	9.77 ± 0.87 ^e^	29.96 ± 0.72 ^c^
Valine (V)	41.97 ± 1.41 ^a^	22.72 ± 0.34 ^d^	33.05 ± 0.60 ^b^	27.73 ± 0.54 ^c^	18.65 ± 1.03 ^e^	10.40 ± 0.17 ^f^	24.00 ± 0.32 ^d^
Methionine (M)	15.57 ± 0.60 ^a^	3.27 ± 0.10 ^c,d^	5.04 ± 0.045 ^b^	3.29 ± 0.90 ^c^	3.16 ± 0.30 ^c,d^	1.31 ± 0.01 ^e^	2.20 ± 0.18 ^d,e^
Cystine (C)	2.45 ± 0.10 ^a^	1.17 ± 0.01 ^c^	1.52 ± 0.25 ^b^	0.00 ± 0.00 ^e^	1.40 ± 0.13 ^b^	0.33 ± 0.06 ^d^	0.43 ± 0.01 ^d^
Isoleucine (I)	52.36 ± 3.42 ^a^	10.90 ± 0.20 ^b^	13.54 ± 0.30 ^b^	11.54 ± 1.10 ^b^	10.29 ± 0.12 ^b^	4.25 ± 0.41 ^c^	9.73 ± 0.15 ^b^
Leucine (L)	41.97 ± 2.20 ^b^	32.41 ± 0.31 ^d^	36.34 ± 2.10 ^c,d^	38.13 ± 0.81 ^b,c^	26.16 ± 1.72 ^e^	12.40 ± 2.01^f^	48.22 ± 2.20 ^a^
Phenylalanine (F)	28.68 ± 1.37 ^a^	10.46 ± 0.30 ^d^	17.42 ± 0.041 ^b^	13.70 ± 1.45 ^c^	10.00 ± 1.28 ^d^	4.73 ± 0.05 ^e^	9.03 ± 0.40 ^d^
Glycine (G)	140.63 ± 1.33 ^a^	107.00 ± 2.02 ^c^	120.48 ± 4.10 ^b^	110.75 ± 1.50 ^c^	53.47 ± 2.40 ^d^	38.83 ± 3.53 ^e^	125.58 ± 3.52 ^b^
Alanine (A)	65.09 ± 1.50 ^a^	50.98 ± 0.87 ^c^	57.27 ± 1.71 ^b^	53.25 ± 0.80 ^c^	27.6 ± 1.52 ^d^	18.09 ± 0.70 ^e^	58.99 ± 1.60 ^b^
Proline (P)	59.77 ± 1.47 ^a^	48.86 ± 0.90 ^c^	54.64 ± 0.75 ^b^	50.36 ± 2.10 ^c^	27.92 ± 0.46 ^d^	16.91 ± 1.38 ^e^	57.60 ± 1.41 ^a,b^
Total amino acid	878.91 ± 30.90 ^a^	579.93 ± 12.40 ^c^	668.9 2± 10.45 ^b^	629.60 ± 12.66 ^b^	402.10 ± 23.24 ^d^	205.52 ± 15.92 ^e^	625.71 ± 6.51 ^b,c^
Hydrophobic AA	388.72	238.91	285.66	258.39	150.73	90.25	278.18
Hydrophilic AA	274.32	194.17	216.7	198.39	148.04	66.53	193.15
Positively charged AA	120.47	69.05	81.47	79.2	49.98	21.97	67.13

* Each value in the table represents the means ± SD of duplicate determinations; ^a−f^ Means with the different superscript letters in the same row indicated significant difference within the hydrolysates (*p* < 0.05).

Relative IC_50_ of enzyme-generated hydrolysates was determined and the lower IC_50_ value indicates higher effectiveness. The alcalase-generated hydrolysates revealed the lowest relative IC_50_ with a value of 1.50 mg/mL. The corresponding values for bromelain, trypsin, papain, pepsin and flavourzyme hydrolysates were 1.73, 2.04, 2.18, 2.31, and 2.54 mg/mL, respectively. All hydrolysates showed significantly (*p* < 0.05) higher IC_50_ values compared to captopril (0.004 mg/mL), a synthetic ACE inhibitor, as a positive control. In general, the difference between the IC_50_ values of the hydrolysates can be related to the number and sequence of the amino acids in the peptide chains of the hydrolysates. The presence of hydrophobic (aromatic or branched side chains) amino acid residues at the three *C*-terminal positions is supposed to increase the ACE inhibitory activity of protein hydrolysates [23]. Moreover, the presence of lysine (K) and arginine (R) at the C-terminal contributes to the potency of the ACE inhibitory activity [24].

*A. lecanora-*generated hydrolysates by different proteases showed different amino acid composition (Table 2). The total hydrophobic and positively charged amino acid contents were higher in alcalase and bromelain hydrolysates (Table 2). The variation in ACE inhibitory activity of hydrolysates might be related to the specificity of enzymes to generate peptides with different amino acid residues [25].

Thus, peptides of various sizes are generated as a function of enzyme and hydrolysis time. Moreover, a comparison of the present data with other researches is quite difficult due to the lack of literature on the ACE inhibitory activity of *A. lecanora* hydrolysates, as well as variations in proteolytic conditions. In this study, the IC_50_ values ranged from 1.50–2.54 mg/mL, which were lower than those reported for other marine hydrolysates. The IC_50_ values for oyster, scallop, codfish skin and herring skin were above 10.0 mg/mL [26]. However, the IC_50_ values of the figureurrent study were higher than the IC_50_ values reported for sea cucumber (*Acaudina molpadioidea*) in the range of 0.615–1.975 mg/mL [27], sardine by 0.082 mg/mL, and bonito by 0.029 mg/mL [28] as well as captopril, as an anti-hypertensive synthetic drug with 0.004 mg/mL.

#### 2.3.2. Anti-Oxidative Activities

##### DPPH Radical Scavenging Activity

**Figure 3:**
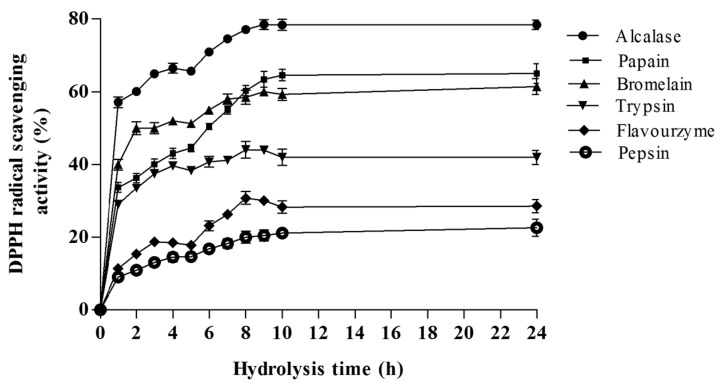
Effect of hydrolysis time on the DPPH radical scavenging activities (%) of different *A. lecanora* hydrolysates generated from enzymatic digestion for 24 h. Sample concentration for this assay was 1.0 mg/mL. Results represent the means ± SD of three replications.

Figure 3 shows the radical scavenging activities of generated hydrolysates at the concentration of 1.0 mg of dry weight/mL. The results revealed that *A. lecanora* showed no DPPH radical scavenging activity before hydrolysis by proteolytic enzymes; however, the radical scavenging activity significantly increased during the hydrolysis time (*p* < 0.05). The DPPH scavenging activities of the hydrolysates varied from 9.00% to 78.56%. The anti-oxidative activities of the hydrolysates generated by alcalase, papain, bromelain, trypsin, flovourzyme and pepsin were 78.56, 65.78, 61.40, 44.10, 30.73 and 22.54%, respectively. The alcalase protein hydrolysate showed the highest radical scavenging activity and the hydrolysates prepared from pepsin and flavourzyme showed the lowest (*p* < 0.05).The obtained results indicate that *A. lecanora* hydrolysates possibly contain peptides with effective proton donor that could react with unstable DPPH free radicals to convert them to more stable products and terminate the radical chain reaction [29]. The effect of the extent of enzymatic hydrolysis on the DPPH scavenging activity was determined (Figure 3). The radical scavenging activity curve pattern showed a sharp increase over the first h of hydrolysis in all treatments except for pepsin and flavourzyme hydrolysates. Then, it increased slowly until reaching a steady-state phase after about 9 h of hydrolysis (Figure 3). Therefore, prolonged hydrolysis time had a positive effect on the DPPH radical scavenging activity. This is in line with previous reports suggesting the increase of DPPH radical scavenging activity is due to the extension of the hydrolysis time [30]. This increase in scavenging activity after hydrolysis can be related to the increase in the solubility of the peptides, generation of active peptides and release of free amino acids.

Therefore, the differences in the radical scavenging properties among hydrolysates might be related to their different peptide size and compositions due to the specificity of the proteolytic enzymes, hydrolysis time and conditions. Higher anti-oxidative activities of alcalase hydrolysates than that of other hydrolysates derived from marine sources have been reported [31]. They found that the anti-oxidative activity of alcalase hydrolysates was higher than pepsin and flavourzyme hydrolysates. The highest activity of alcalase might be related to its action as an endo-peptidase. Alcalase cleaves peptide bonds at the interior of the polypeptide chain, and generates small and medium-sized oligopeptides or polypeptides, some of which show anti-oxidative activity [32]. Furthermore, it has been indicated that the DPPH radical scavenging activity is related to the amino acid composition [33]. It is believed that the aromatic amino acids (tyrosine, histidine, tryptophan and phenylalanine), hydrophobic amino acids (valine, leucine, and alanine), and methionine play a crucial role in the DPPH radical scavenging activity [34]. Thus, the presence of these amino acids in a peptide sequence might increase its access to reactive free radicals more easily to generate the anti-oxidative activity [35]. The difference in the amino acid composition of *A. lecanora* hydrolysates derived from different proteases might be related to the specificity of the enzymes (Table 2). In this regard, the concentrations of aromatic and hydrophobic amino acids in alcalase-generated hydrolysates were higher than other hydrolysates, which could explain its interesting anti-oxidative activity. 

**Figure 4:**
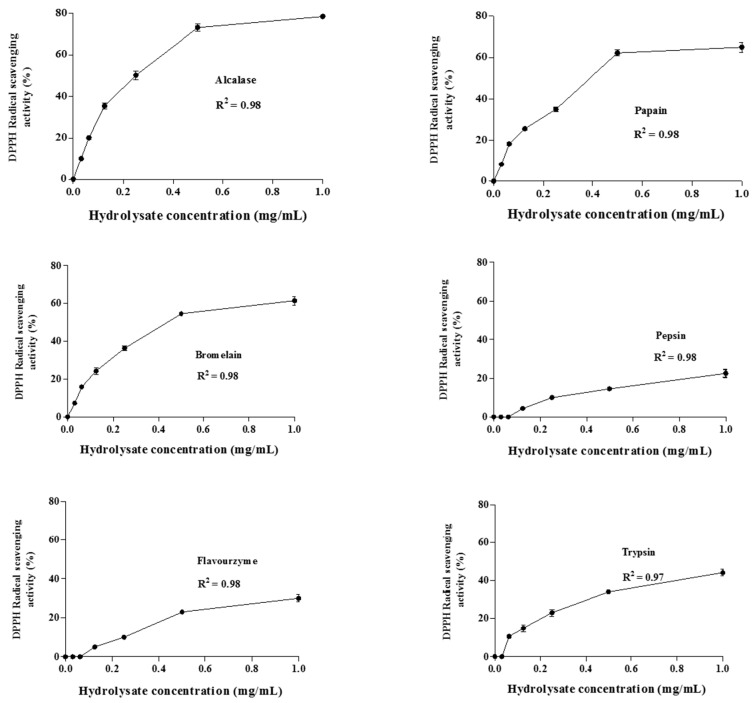
Changes in DPPH radical scavenging activities (%) of *A. lecanora* hydrolysates as a function of concentrations between 0.0 and 1.0 mg dry weight/mL. Each value is the mean ± SD of three replications.

Figure 4 shows the changes in the radical scavenging activity of the hydrolysates as a function of concentration (0.0–1.0 mg of dry weight/mL). The results demonstrated that the DPPH radical scavenging activities increased as the concentration of all hydrolysates was increased. In the current study, glutathione was used as a positive control since it is recognized as a potent anti-oxidative peptide [34]. The lowest relative IC_50_ value was obtained from alcalase (0.181 mg/mL) and papain (0.194 mg/mL) hydrolysates, followed by bromelain (0.20 mg/mL), flavourzyme (0.32 mg/mL), pepsin (0.34 mg/mL) and trypsin (0.51 mg/mL). All hydrolysates exhibited higher IC_50_ than that obtained with glutathione as a positive control (0.106 mg/mL) at the same concentration. This was in line with Jia *et al.* [15] who reported that the DPPH radical scavenging activity of the alcalase pollack skin hydrolysate was lower than that of glutathione. The results revealed that the *A. lecanora* hydrolysates, generated from alcalase and papain were found to possess strong radical scavenging activities compared to other marine sources such as marine rotifer (46–50% at 1 mg/mL) [36], fish protein (*Catla catla*) (64.65% at 2 mg/mL) [37], bluefin tuna (*Thunnus thynnus*) heads (87% at 3 mg/mL) [38], and pollack skin hydrolysate (IC_50_ of 2.5 mg/mL) [15].

##### Ferrous Ion Chelating Activity (FIC)

Trace metal ions such as Fe^2+^ can catalyze the formation of reactive oxygen species, such as hydroxyl radical and superoxide anion. In particular, Fe^2+^ generates hydroxyl radicals by decomposing lipid hydroperoxides through the Fenton reaction. Potentially, these free radicals contribute to some diseases related to oxidative stress. Moreover, the excess of iron could cause toxicity in body organs especially the liver. Therefore, the chelating of these metal ions by using anti-oxidative peptides retards the oxidation reaction [38].

**Figure 5:**
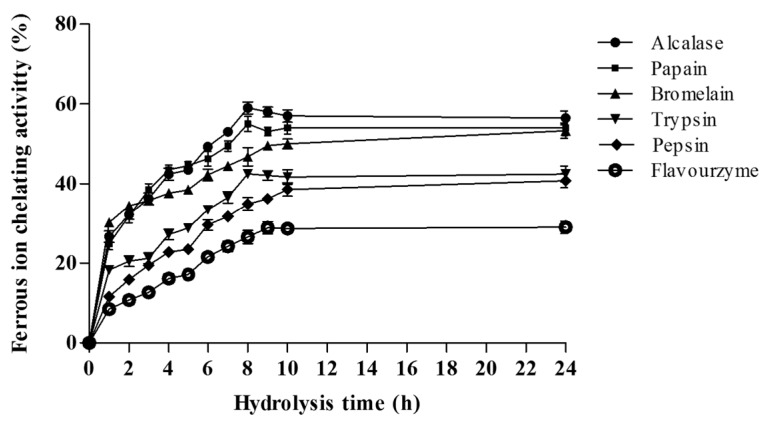
Effect of hydrolysis time on the ferrous ion chelating activity (FIC) (%) of *A. lecanora* hydrolysates generated from enzymatic digestion for 24 h. Sample concentration for this assay was 1.0 mg dry weight/mL. Each value is the mean ± SD of three replications.

Ferrous ions (Fe^2+^) chelating activities of the *A. lecanora* hydrolysates at 1.0 mg of dry weight/mL were determined by measuring the inhibition of the Fe^2+^-ferrozine complex formation. The results are expressed as relative iron chelating activity compared with the unchelated Fe^2+^ reaction (Figure 5). 

As displayed in Figure 5, the FIC of alcalase, papain, bromelain and trypsin hydrolysates increased dramatically over the first h of hydrolysis, followed by a slow rise, and reached maximum activity after 8 h of hydrolysis. The maximum FIC activity was observed in the bromelain-generated hydrolysate after 24 h hydrolysis. However, the Fe^2+^ chelating activities of the hydrolysates prepared by pepsin and flavourzyme increased gradually during hydrolysis, until reaching a steady state phase after 24 h. Thus, metal-chelating activity could be increased through hydrolysis with certain proteolytic enzymes and by increasing the hydrolysis time. In this regards, Yea *et al.* [10] reported that prolonged hydrolysis resulted in high metal-chelating activity.

The highest Fe^2+^chelating activity was achieved by alcalase-generated hydrolysates with a value of 59.00% after 8 h of hydrolysis followed by papain (55.00% after 8 h), bromelain (53.30% after 24 h), trypsin (42.30% after 8 h), pepsin (40.80% after 24 h) and flavourzyme (29.00% after 24 h), respectively. Statistical analysis revealed no significant difference among alcalase, bromelain and papain hydrolysates, whilst the differences in trypsin, pepsin and flavourzyme were significant (*p* < 0.05). Moreover, previous studies on the ferrous ion chelating activity of hydrolysates demonstrated that the activity could be affected by type of protease, nature of protein sources, length of hydrolysis, concentration and amino acid composition in the peptide sequences [38]. Thus, hydrolysisis is considered to be an effective way to generate peptides with anti-oxidative activity in terms of radical scavenging and ferrous ion chelating activities.

A wide range of Fe^2+^chelating activity for alcalase-generated hydrolysates derived from different marine sources with various concentrations has been reported. Foh *et al.* [39] reported that alcalase hydrolysates derived from tilapia fish showed a high chelating activity by a value of 82.50% at 5 mg/mL compared to flavourzyme and neutrase hydrolysates, which had chelating activities of 75.80% and 77.23%, respectively. 

**Figure 6:**
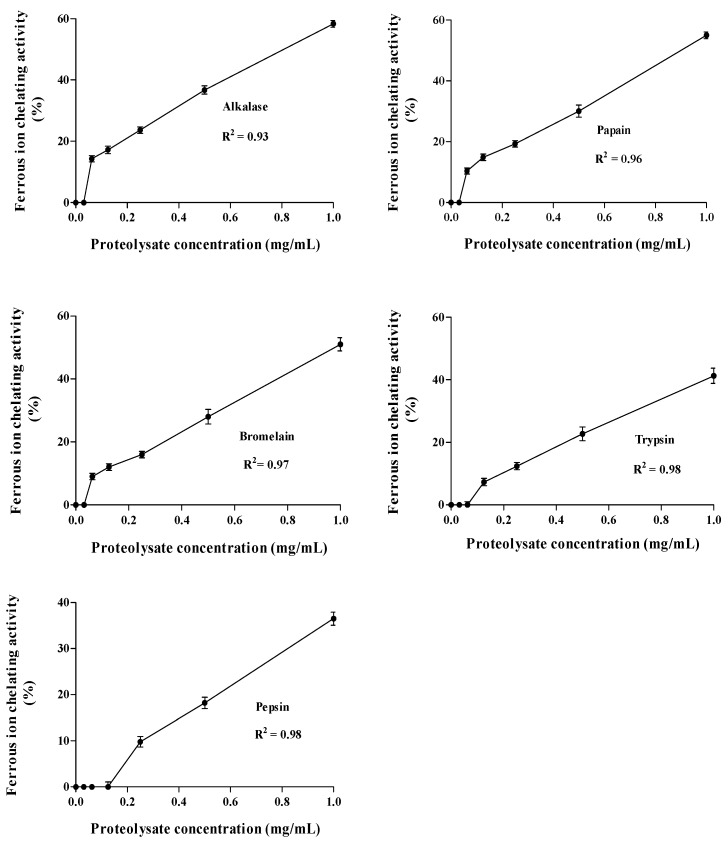
Changes in ferrous ion chelating activities (%) of *A lecanora*/hydrolysates at concentration between 0.0 and 1.0 mg dry weight/mL Each value is the mean ± SD of three replications.

The generated-hydrolysates were selected to determine the effects of powder concentrations from 0.0 to 1.0 mg/mL on the FIC activity (Figure 6) and EDTA-Na_2_ were used as positive control. The proteolytic enzyme generated-hydrolysates exhibited a concentration-dependent manner and their activity increased linearly by increasing concentrations by *R*^2^ > 0.93 (Figure 6). The lowest IC_50_ value was obtained with hydrolysate prepared by alcalase (0.42 mg/mL after 8 h), followed by papain (0.45 mg/mL after 8 h), bromelain (0.46 mg/mL after 24 h), trypsin (0.49 mg/mL after 8 h) and pepsin (0.52 mg/mL after 24 h). All hydrolysates showed significantly (*p* < 0.05) higher IC_50_ compared to EDTA (10.54 µg/mL). 

During hydrolysis, peptide cleavages led to an increase in the concentration of carboxylic (–COOH) and amino groups in the side chains of the acidic and basic amino acids that enhance the chelating activity of hydrolysates [20]. The direct relationship between peptide concentration and increase in the chelating activity that has been already indicated by Saiga *et al.* [40] support this idea. They also conclude that the acidic and basic amino acids might play an important role in Fe^2+^ and Cu^2+^chelation. Thus, the highest Fe^2+^ chelating activity in the peptides after digestion using alcalase was probably due to the presence of the acidic amino acids such as glutamic and aspartic acids, and basic amino acids including lysine and arginine. 

Therefore, the results of this study demonstrated that *A. lecanora* hydrolysates possess potential for use as a functional food source due to their anti-hypertensive and anti-oxidative properties. It can be concluded that the mentioned activities of *A. lecanora* hydrolysates were strongly affected by the type of enzyme and hydrolysis duration. Alcalase specificity is much more appropriate to the available cutting sites of the *A. lecanora* protein and this resulting mixture of peptides showed the highest ACE inhibitory and anti-oxidative activities compared to other hydrolysates.

### 2.4. Correlation between ACE Inhibitory and Anti-Oxidative Activities of A. lecanora Alcalase-Generated Hydrolysates over 24 h of Hydrolysis

**Figure 7:**
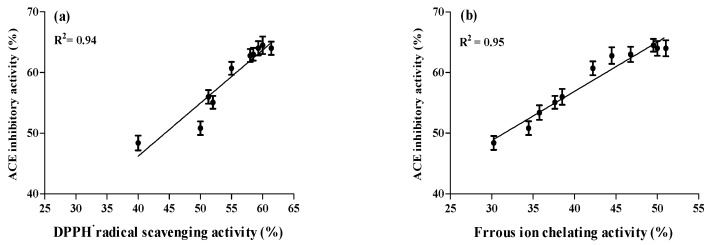
Correlation between bioactivities (%) of *A. lecanora* alcalase hydrolysates. (**a**) ACE inhibition *versus* DPPH radical scavenging activity; (**b**) ACE inhibition *versus* metal ion chelating activity. *R*^2^ values indicated the best-fit linearity functions. Bars represent standard deviations from triplicate determinations.

Hypertension and oxidative stress are two major causes of cardiovascular diseases. In the condition of high blood pressure, angiotension II increases the oxidative stress as it intervenes with several of its cellular actions through stimulating the formation of intracellular reactive oxygen species (ROS) [8]. Therefore, apart from control of blood pressure, ACE inhibitors have been shown to increase the anti-oxidative defense system through inhibition of the formation of angiotensin II [41]. Figure 7 reveals that all peptide fragments with ACE inhibition displayed anti-oxidative activities over 24 h of proteolysis. Significant Pearson correlation coefficients were observed among ACE inhibitory and anti-oxidative properties. The correlations between ACE, FIC and DPPH radical scavenging activities were 0.95 and 0.94, indicating a strong positive correlation. Therefore, the anti-oxidative activities increased by increasing the ACE inhibitory activities, suggesting that generated peptides with similar structure can exhibit dual bioactivities with ACE inhibitory and anti-oxidative properties. These findings are in accordance with Yea *et al.* [10].

## 3. Experimental Section 

### 3.1. Raw Material

Fresh samples of *Actinopyga lecanora* were purchased from Pantai Merdeka in the Kedah state, Malaysia, and transported on ice to the laboratory within 24 h. Upon arrival, the internal organs were removed and samples were rinsed with cold distilled water, packed in a polyethylene plastic bags and stored in a freezer at −80 °C (Ultra-Low Temperature Freezer, Eppendorf, Hamburg, Germany) until further use.

### 3.2. Chemicals

Alcalase^®^2.4 L from *Bacillus licheniformis* and flavourzyme^®^ were obtained from Novoenzyme (Bagsvaerd, Denmark). Bromelain and papain from papaya were obtained from Acros Organics Co. (St. Louis, MO, USA). Pepsin from porcine gastric mucosa was supplied by Merck Co. (Darmstadt, Germany), and trypsin from beef pancreas was supplied by Fisher Scientific (Atlanta, GA, USA). *o*-phtaldialdehyde (OPA) was purchased from Sigma-Aldrich (Munich, Germany). 2,2-Diphenyl-1-Picrylhydrazyl (DPPH), Sodium tetraborate was purchased from Sigma Chemical Co. (St. Louis, MO, USA). Glutathione and ferrozine were purchased from Acros Organics Co. (St. Louis, MO, USA). Hippuryl-histidyl-leucine (HHL), captopril and angiotensin converting enzyme (ACE) derived from rabbit lung were purchased from Sigma Chemical Co. (St. Louis, MO, USA). 

### 3.3. Preparation of Enzymatic Hydrolysates from A. lecanora

Prior to enzymatic hydrolysis, the freeze-dried *A. lecanora* was ground into a powder using a Warring blender (model 32 BL 79, Warring, Winsted, Winchester, CT, USA) and passed through a #35 mesh sieve (600 μm) to obtain milled whole *A. lecanora.* The sample (10 g) was mixed with 50 mL distilled water and dialyzed in 12–14 kDa molecular weight cut off dialysis tube according to the manufacturer’s guide (Visking, 28.6 mm diameter). The tubes were immersed in an appropriate buffer solution (50 mM) for 24 h at 4 °C. After dialysis, the sample was hydrolyzed independently with each of the papain (phosphate buffer, pH 7, 60 °C), alcalase (borate buffer, pH 8, 37 °C), pepsin (tris-HCL buffer, pH 1.5, 37 °C), trypsin (borate buffer, pH 8, 37 °C), flavourzyme (phosphate buffer, pH 7, 55 °C) and bromelain (acetate buffer, pH 5, 55 °C) at a ratio of 1:100 (enzyme/substrate *w*/*w*). Proteolysis was carried out for 24 h in a water-bath with continuous stirring at 150 rpm. The enzyme was re-added every 5 h during the proteolysis. Samples were withdrawn before hydrolysis as a control and at 1 h intervals during hydrolysis process up to 24 h. The enzymatic reaction was immediately terminated by heating the samples in a boiling-water bath for 15 min to inactivate the proteases. After centrifugation (10,000× *g*, 20 min at 4 °C), the resulting supernatant containing peptides was collected and used for determination of ACE inhibitory and anti-oxidative activities.

### 3.4. Peptide Content Measurement

Peptide content was measured using the O-phthaldialdehyde method (OPA) [42] with some modifications [43]. Sample (36 µL) and OPA solution (270 µL) were pipetted into individual wells using a 96-well plate reader. The mixture was incubated for 2 min at room temperature and the absorbance was measured at 340 nm. To calculate the peptide content a glutathione calibration curve was constructed in the range of 0.01–0.25 mg/mL. The test was carried out in triplicate and peptide content was expressed as mg glutathione per mL of hydrolysates.

### 3.5. Amino Acid Composition

The amino acid composition was determined using the Khan method [44]. Freeze-dried samples were hydrolyzed by using 6 N hydrochloric acid (HCl) at 110 °C for 24 h. Upon completion, the l-α-amino-*n*-butyric acid (AABA) as an internal standard was added to the hydrolyzed samples and then made up to 50 mL using de-ionized water. The internal standard α-aminobutyric acid was added to the hydrolyzed samples and filtered was through a filter paper (Whatman No. 1). Ten-microlitre aliquots of a sample or 10 μL of the amino acids standard mixture was dried under vacuum (37 °C, 20 mm Hg) for 30 min in a vial. The dried sample or standard was dissolved in a 20 μL of a solution consist of methanol, water and triethylamine (2:2:1 *v*/*v*), and after swirling immediately dried under vacuum (Rhino Pump, Ningbo, China) for 30 min. After drying, the samples were derivatized using 20 μL of a reagent comprised of methanol, triethylamine, water and phenylisothiocyanate (PITC) (7:1:1:1 *v*/*v*). After mixing, the samples were allowed to stand at room temperature for 20 min, followed by vacuum drying for 30 min. The derivatized samples were kept at −80 °C until analysis. A 20 µL of the derivatized sample was injected into the HPLC system equipped with a multi-wavelength detector (MD-2010 plus), 2 pumps (PU-2080 plus), and an online degasser (DG-2080-54) (Jasco, Tokyo, Japan). The amino acids were separated by gradient elution using the two mobile phase on a Purospher STAR RP-18e column (5.0 µm, 250 mm × 4.6 mm, Merck, Darmastadt, Germany) with the temperature controlled at 43 °C and a flow rate set at 1 mL/min. The mobile phase consisting of buffer A ammonium acetate (0.1 M) ammonium, pH 6.5) and buffer B (0.1 Mammonium acetate containing acetonitrile, methanol, (44:46:10 *v*/*v*, pH 6.5) The UV absorption detector at a wavelength of 254 nm was employed to monitor amino acids. The amount of amino acids was calculated, based on the peak area in comparison with that of a standard. 

### 3.6. ACE Inhibitory Activity 

ACE assay was performed using the method that is described by Jimsheena & Gowda [45] with some modifications. The assay mixture contained 0.125 mL of 0.1 M sodium borate buffer (pH 8.3) containing 0.3 M NaCl, 50 μL of 5 mM HHL, 10 μL of ACE enzyme and 10 μL of sample. The reaction was terminated after incubation at 37 °C for 60 min, through the addition of 75 μL of 1M HCl. After stopping the reaction, 150 μL of pyridine was added followed by 75 μL of benzene sulphonylchloride (BSC) and the solution was mixed before cooling down on ice. Once cooled, 200 μL solution was transferred to the 96-well plate. The absorbance was measured at 410 nm using a 96-plate reader.The experiments were conducted in triplicates. The following equationwas applied to calculate the ACE inhibition.

ACE inhibition (%) = [(B − A)/(B − C)] × 100
(1)

where B is the absorbance with ACE and HHL without the ACE inhibitor component; A is the absorbance with ACE, HHL and C is the absorbance with HHL without ACE and ACE inhibitor components.

### 3.7. DPPH Free Radical Scavenging Assay

The DPPH free radical scavenging activity was determined according to the method described by Hwang *et al.* [46] with some modifications. Briefly, 100 μLof DPPH solution (0.1 mM in 80% ethanol) was mixed with 100 μL of sample solution in 96-well plate. The mixtures were incubated for 30 min in a dark condition at room temperature and the reduction of DPPH was measured at 517 nm using the 96-well plate reader (Power Wave X340, BioTek instruments, INC, Winooski, VT, USA). Glutathione was used as reference standard and the following equation was used to determine the scavenging activity (%). The tests were carried out in triplicate.

DPPH radical scavenging activity (%) = [(A_control_ − A_sample_)/A_control_] × 100 (2)

### 3.8. Ferrous Ion-Chelating Activity

The ferrous ion-chelating activity was determined according to the method described by Wang *et al.* [47]. Sample solution (100 µL) was mixed with 135 µL of distilled water and 5 µL of 2 mM FeCl_2_. The reaction was initiated by addition of 10 µL of 5 mM ferrozine. After incubation for 10 min at room temperature, the absorbance was measured at 562 nm using a 96-well plate reader. Distilled water (100 µL) instead of sample solution was used as a control (A_0_). Distilled water (10 µL) instead of ferrozine solution was used as a blank (A_2_) and A_1_ is the absorbance of sample and reference standard (EDTA-Na_2_). The ferrous ion-chelating ability was determined using the following equation:

Ferrous ion-chelating ability (%) = [A_0_ − (A_1_ − A_2_)/A_0_] × 100 (3)

### 3.9. IC_50_ Determination of the Hydrolysates

The IC_50_value is defined as the concentration of hydrolysates that is able to inhibit half-maximal of the ACE and oxidation activities. Different concentrations of hydrolysates were selected and evaluated for their ACE inhibitory (%) and anti-oxidative (%) activities. The IC_50_ of the different hydrolysates was determined by plotting the ACE inhibition (%) and anti-oxidative (%) activities against the various concentrations of hydrolysates. The IC_50_ of the peptides were compared with the IC_50_ of captopril, glutathione and Na_2_EDTA as positive standards. Experiments were done in triplicate.

### 3.10. Statistical Analysis

The data obtained were subjected to one-way analysis of variance. Tukey’s test was performed to determine the significant differences at the 5% probability level.

## 4. Conclusions

The use of enzymatic hydrolysis for generating *A. lecanora* hydrolysate with dual bioactivities of ACE inhibitory and anti-oxidative activities is feasible. The results demonstrated that the type of enzyme and duration of hydrolysis greatly influenced the amino acid residue composition and the resulting ACE inhibitory and anti-oxidant activities. Among the different proteases tested, alcalase was found to be the most efficient for generation of hydrolysates with the highest ACE inhibitory and anti-oxidative activities. The dual bioactivities of *A. lecanora* hydrolysates as a rich source of bioactive peptides may be harnessed for cardiovascular health-related diseases. Further investigations are necessary to purify and identify the individual peptides responsible for ACE-inhibitory and anti-oxidant activities in *A. lecanora* hydrolysates. 
